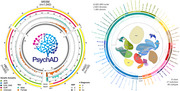# Single‐cell atlas of transcriptomic vulnerability across multiple neurodegenerative and neuropsychiatric diseases

**DOI:** 10.1002/alz70855_097915

**Published:** 2025-12-23

**Authors:** Donghoon Lee

**Affiliations:** ^1^ Icahn School of Medicine at Mount Sinai, New York, NY, USA

## Abstract

**Background:**

Neurodegenerative and neuropsychiatric diseases impose a significant societal and public health burden. However, our understanding of the molecular mechanisms underlying these highly complex conditions remains limited.

**Method:**

To gain deeper insights into the etiology of different brain diseases, we used specimens from 1,494 unique donors to generate a population‐scale single‐cell transcriptomic atlas of the human dorsolateral prefrontal cortex (DLPFC), comprising over 6.3 million individual nuclei. The cohort includes neurotypical controls as well as donors affected by eight common and complex brain disorders: Alzheimer's disease (AD), diffuse Lewy body disease (DLBD), vascular dementia (Vas), Parkinson's disease (PD), tauopathy, frontotemporal dementia, schizophrenia, and bipolar disorder.

**Result:**

We show that inter‐individual variation accounts for a substantial portion of gene expression variation in the DLPFC. By comparing transcriptomic variation across diseases, we reveal universal signatures enriched in basic cellular functions such as mRNA splicing and protein localization. After discounting these cross‐disease signatures, we show strong genetic and transcriptomic concordance among AD, DLBD, Vas, and PD, largely driven by alteration of synaptic signaling functions in neurons. Furthermore, we characterize transcriptomic variation among different AD phenotypes that were distinct from healthy aging. We uncover mitigating effects of interneurons and aggravating effects of immune and vascular cells in AD dementia. Further exploring the effect of the neuropsychiatric symptoms frequently accompanying AD, we identify a link to deep layer excitatory neurons. By constructing transcriptome trajectories that capture AD progression, we show cell‐type specific responses implicated in early and late stages of AD.

**Conclusion:**

Our atlas provides an unprecedented perspective of the transcriptomic landscape in neurodegenerative and neuropsychiatric diseases, shedding light on shared and distinct processes involving the neuro‐immune‐vascular systems, and identifying potential targets for therapeutic intervention.